# The effect of preoperative PC6 intradermal acupuncture combined with dexamethasone and palonosetron on preventing postoperative nausea and vomiting in bilateral orthognathic surgery: a research protocol for blind, double-dummy, randomized controlled clinical trial

**DOI:** 10.3389/fmed.2026.1774071

**Published:** 2026-04-07

**Authors:** Xianghui Xu, Changxun Yin, Jiachun Cai, Jiexiong Li, Jianjin Li, Zhitao Li

**Affiliations:** 1 Department of Pain, Peking University Shenzhen Hospital, Shenzhen, China; 2 Department of Anesthesiology, Peking University Shenzhen Hospital, Shenzhen, China; 3 Shenzhen Eye Hospital, Shenzhen, China

**Keywords:** acupuncture, intradermal needle, orthognathic surgery, PC6 (“Neiguan”), postoperative nausea and vomiting

## Abstract

**Background:**

Pain and postoperative nausea and vomiting (PONV) are the most prevalent postoperative complications following orthognathic procedures. Acupuncture therapy is advocated as one of the treatment alternatives for preventing PONV. This trial aims to investigate the efficacy of preoperative intradermal acupuncture in combination with dexamethasone and palonosetron in high-risk patients undergoing bimaxillary orthognathic surgery, employing a multimodal risk-reduction strategy aligned with consensus guidelines.

**Methods:**

This single-center, double-blind, randomized controlled trial will enroll 216 participants allocated to two groups: group A, the acupoint stimulation group and group B, the sham stimulation group. All subjects will receive standardized PONV prophylaxis including dexamethasone 5 mg IV, palonosetron 0.075 mg IV, target-controlled propofol infusion (0.5 μg/mL), and laryngeal packing following anesthesia induction. Patients in both groups receive two sterile thumb-tack needles – one real needle and one identical-appearing sham needle without a pointed tip. In group A, the real needles are placed at the PC6 acupoint, and the sham needles are placed at the sham acupoint on the forearm. In group B, the real needles are placed at the sham acupoint, while the sham needles are placed on the PC6 acupoint. Finally, all patients will have the sterile opaque patches applied to each acupoint for allocation concealment. The needles will remain in place up to 24 h postoperatively at least. The primary outcome will be the incidence of PONV at 24 h postoperatively. Secondary outcomes will include the incidence rates of PONV at different postoperative intervals and a 15-point quality of life recovery score at 24 h postoperatively.

**Discussion:**

Despite dual-mechanism pharmacological prophylaxis, orthognathic surgery patients exhibit persistent postoperative nausea rates approaching 50%. It is necessary to introduce a third combined measure to prevent PONV. This trial aims to investigate a novel triple-modality prophylactic strategy for PONV mitigation in bimaxillary orthognathic procedures.

**Systematic review registration:**

https://itmctr.ccebtcm.org.cn/mgt/project/view/1966089260633292800, Identifier ITMCTR2025001713.

## Background

Orthognathic surgery can effectively address malocclusion and optimize facial symmetry aesthetics, occlusal patterns, and temporomandibular joint dynamics through three-dimensional correction of malocclusions of the jaw and face. With the increasing demand for psychosocial well-being enhancement, particularly in social interaction domains, more and more candidates pursue bimaxillary surgery (1). The most common postoperative complications of orthognathic surgery are pain and PONV. Severe PONV exerts a stronger impact on early postoperative recovery than moderate pain. PONV is a significant factor affecting overall perioperative satisfaction (2). PONV can lead to gastrointestinal reactions, electrolyte and acid–base imbalances, and in severe cases, may result in wound dehiscence, sudden increases in blood pressure and intracranial pressure, and aspiration pneumonia (3). In bimaxillary patients, mouth opening is limited due to elastic steel wire fixation, external bandage fixation, and facial swelling. PONV may cause fatal aspiration (4).

Female gender, younger age, non-smokers, a history of motion sickness, laparoscopic surgery, inhalation anesthesia administration, and high-dose opioid utilization constitute well-established independent predictors of PONV (5). The incidence of PONV in general surgical populations approximates 30%, while it can be as high as about 60% in orthognathic surgery patients, despite routine use of dexamethasone and 5-HT3 antagonists for prevention (6–8). Intraoperative controlled hypotension, rapid palatal expansion, elastic traction between the upper and lower jaws, intermaxillary fixation wires, and blood ingestion postoperatively are independent risk factors that distinguish orthognathic surgery from other general surgical procedures (9–11). Notably, bimaxillary osteotomy emerges as the most significant predictor of PONV development, necessitating distinct prophylactic strategies compared with general surgical procedures. Although the consensus guidelines recommend using propofol for induction/maintenance as strategies to reduce baseline PONV risk, current evidence suggests total intravenous anesthesia (TIVA) demonstrates may not fully address delayed PONV in orthognathic settings, potentially attributable to the temporal pattern of PONV manifestation (12). Whereas inhalational agent-induced emesis predominantly occurs within the immediate postoperative phase (0–2 h) (13), maxillofacial surgery patients exhibit 2.7-fold higher PONV incidence during the delayed recovery window (2–24 h postoperatively) (14).

For the aforementioned high-risk factors, various antiemetic regimens with multimodal pharmacological interventions targeting distinct pathophysiological pathways are currently incorporated in orthognathic surgery, such as serotonin 3 (5-HT3) receptor antagonists, glucocorticoids, opioid-sparing analgesic protocols, nasogastric decompression, and throat packs. These interventions exhibit distinct pharmacokinetic profiles, therapeutic efficacy, and adverse effect spectra, with no monotherapy demonstrating complete PONV prevention (15). Combination therapy is superior to monotherapy. In current anesthesia practice, dexamethasone combined with palonosetron, a second-generation 5-HT3 receptor antagonist with prolonged receptor-binding kinetics (mean elimination half-life: 40 h), constitutes the baseline dual antiemetic regimen for high-risk patients (16, 17). Acupuncture therapy is also endorsed as one of options for preventing PONV in the Fourth Consensus Guidelines for the Management of PONV in 2020 (3). Commonly used acupoints include the Neiguan point (PC6) on the pericardium meridian, the Zusanli point (ST36) on the stomach meridian, and the Hegu point (LI4) on the large intestine meridian. While the precise mechanism remains incompletely elucidated, it may involve modulating sympathetic nerve activity and gastrointestinal function, promoting potential gastrointestinal motility to prevent PONV.

Acupuncture has been well-documented in literatures on abdominal, gynecological, and otolaryngological surgery in PONV prophylaxis. Nevertheless, its application in orthognathic procedures remains limited. Existing acupuncture protocols predominantly involve intraoperative acupoint stimulation that terminates upon patient transfer from the operating theater. It is unclear whether acupuncture can offer comparable benefits in preventing PONV in orthognathic surgery patients, as it does in other abdominal surgeries. Current evidence derives primarily from trials comparing acupuncture monotherapy against single-agent pharmacotherapy, notably omitting guideline-recommended combination prophylaxis in control arms – a methodological limitation that reduces external validity and impedes clinical implementation (18–20).

Based on the risk minimization strategy, we evaluate the effect of preoperative intradermal acupuncture in PC6 point combined with dexamethasone and palonosetron in preventing PONV of the patients scheduled for bilateral orthognathic surgery, to verify the incremental antiemetic benefits of acupoint stimulation beyond routine PONV prevention strategies.

## Methods/design

### Study design

This trial is a single-center, prospective, randomized, double-blind, double-mimicry placebo-controlled trial, and will be conducted in Peking University Shenzhen Hospital from October 2025 to March 2028. The protocol has been approved by the Medical Ethics Committee of Peking University Shenzhen Hospital (approval number: 2025124) and registered at the ITMCTR (Trial registration number: ITMCTR2025001713).

### Subject evaluation, screening and recruitment process

**Figure 1:**
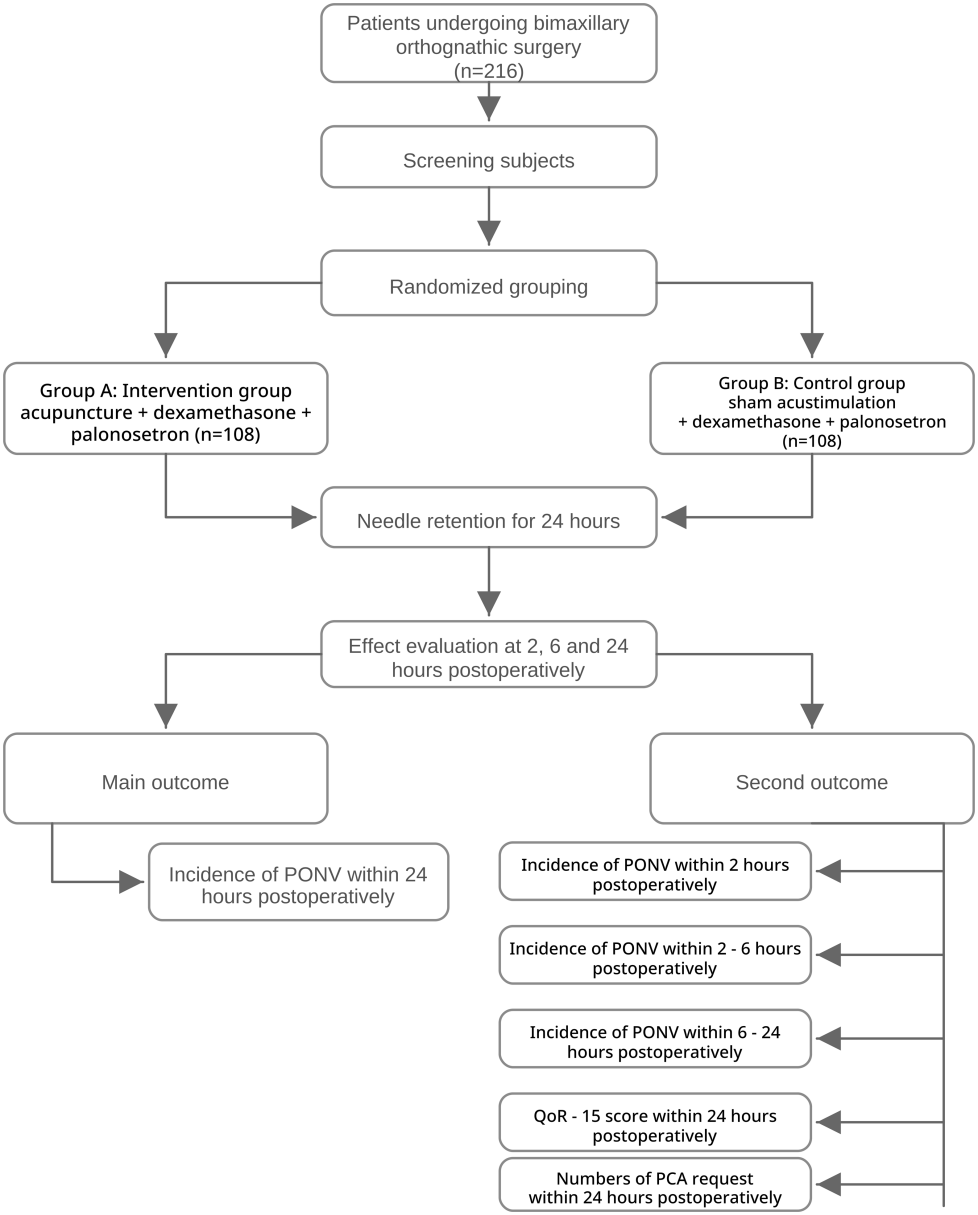
Protocol flow. PONV, postoperative nausea and vomiting; QoR-15 score, Quality of Recovery score; PCA, patient-controlled analgesia.

A total of 216 patients scheduled for elective bilateral orthognathic surgery will be evaluated, screened, and sequentially recruited by the attending anesthesiologists one day prior to the surgical procedure. Eligible participants will be randomly assigned in a 1:1 ratio to either group A (acupoint stimulation group) or group B (sham stimulation group). Informed consent must be obtained from all participants before randomization. The study protocol flowchart is presented in Figure 1, while the detailed study schedule is summarized in Table 1.

**Table 1:** Study schedule.

	Screening	Intervention	Operation	Recovery	Follow-up		
Time	The day before operation	Before induction	During surgery	Stay in the recovery room	0–2 h Postoperative	2-6 h Postoperative	6-24 h Postoperative
Eligibility screen	✓						
Informed consent	✓						
Demographic data	✓						
ASA status	✓						
Apfel score	✓						
Randomisation	✓						
PC6 stimulation		✓	✓	✓	✓	✓	✓
Duration of surgery			✓				
Duration of anesthesia			✓				
Duration of controlled hypotension			✓				
Consumption of narcotic drugs			✓				
Extubation time				✓			
NRS score right after extubation				✓			
Duration of PACU stay				✓			
Nausea				✓	✓	✓	✓
Vomiting				✓	✓	✓	✓
Numbers of PCA request							✓
Total PCA volume (mL) delivered							✓
QoR-15 assessment							✓

ASA status, American Society of Anesthesiologists status; NRS, Numerical Rating Scale; PACU, post-anesthesia care unit; PONV, postoperative nausea and vomiting; PCA, patient-controlled analgesia.

### Eligibility criteria

The inclusion criteria are defined as follows: (1) Patients scheduled for bilateral orthognathic surgery (including Le Fort I osteotomy and bilateral sagittal split osteotomy); (2) male and female patients aged 18 to 64 years; (3) American Society of Anesthesiologists (ASA) physical status I-II; (4) patients informed about the study protocol with signed written consent.

The exclusion criteria comprise: (1) Presence of local scars or infections in acupuncture areas; (2) limb absence or peripheral nerve injury; (3) body mass index (BMI) > 30 kg/m^2^; (4) history of substance abuse; (5) history of central nervous system disorders or psychiatric conditions; (6) requirement for postoperative intensive care unit (ICU) transfer; (7) current participation in other clinical trials.

Participants meeting any of the following criteria post-enrollment will be withdrawn: (1) Surgical cancellation or conversion to a single-jaw procedure; (2) inability to tolerate acupuncture therapy; (3) premature removal (<24 h) of thumb-tack needles for any reason. All withdrawal cases must be thoroughly documented. Participants who have received the intervention will complete follow-up assessments. No efficacy analyses will be performed for withdrawn cases.

### The principle of randomness

This study will utilize computer-generated block randomization with a total sample size of 216 cases divided into 54 blocks, each containing 4 cases. These blocks are equally allocated to two groups, with sequences randomly arranged within each block. Allocation details will be secured in opaque, sealed envelopes. After obtaining signed informed consent, the acupuncturist administering the intervention will access the randomization sequence through the Random.org website (https://www.random.org/) and then open the corresponding sealed envelope to access group assignment details.

### Blind method

This trial employs a sham-controlled design. The true stimulation group receives intervention at the PC6 acupoint, while the sham stimulation group undergoes stimulation at a non-acupoint site on the forearm. A designated acupuncturist performs thumb-tack needle insertion at predetermined locations according to group assignment, followed by application of an opaque white sterile patch to secure the device and maintain blinding. The same practitioner removes all thumb-tack needles 24 h post-intervention. Both needle insertion and removal procedures are exclusively performed by the assigned acupuncturist. A trained follow-up coordinator conducts postoperative assessments.

To assess the integrity of participant blinding, within 24 h postoperatively, participants will be asked to guess whether the stimulation was received at a real acupoint (PC6) or at a sham acupoint (non-acupoint). Responses will be recorded on the case report form (CRF), and the proportion of correct guesses will be summarized.

### Emergency unblinding

**Figure 2:**
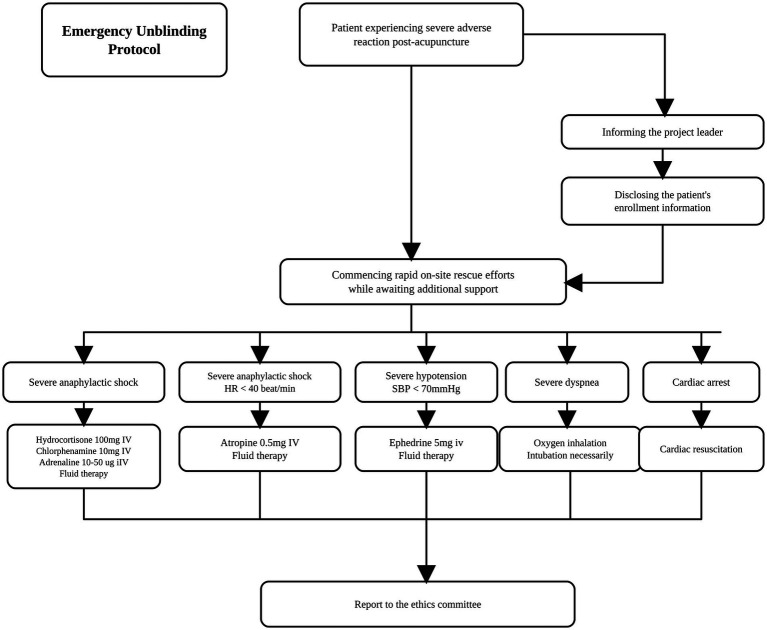
Emergency unblinding protocol. HR, heart rate; SBP, systolic blood pressure; IV, intravenous injection.

Following acupoint stimulation, immediate emergency intervention must be initiated if patients develop acute allergic reactions, severe bradycardia (heart rate <40 bpm), profound hypotension (systolic blood pressure <70 mmHg), acute respiratory distress, or cardiac arrest. Participants meeting these criteria will be immediately withdrawn from the trial. The principal investigator will disclose the allocation details to the treatment team and submit a formal report to the ethics committee after stabilization of the patient’s condition. The emergency unblinding procedure is detailed in Figure 2.

### Intervention

**Figure 3:**
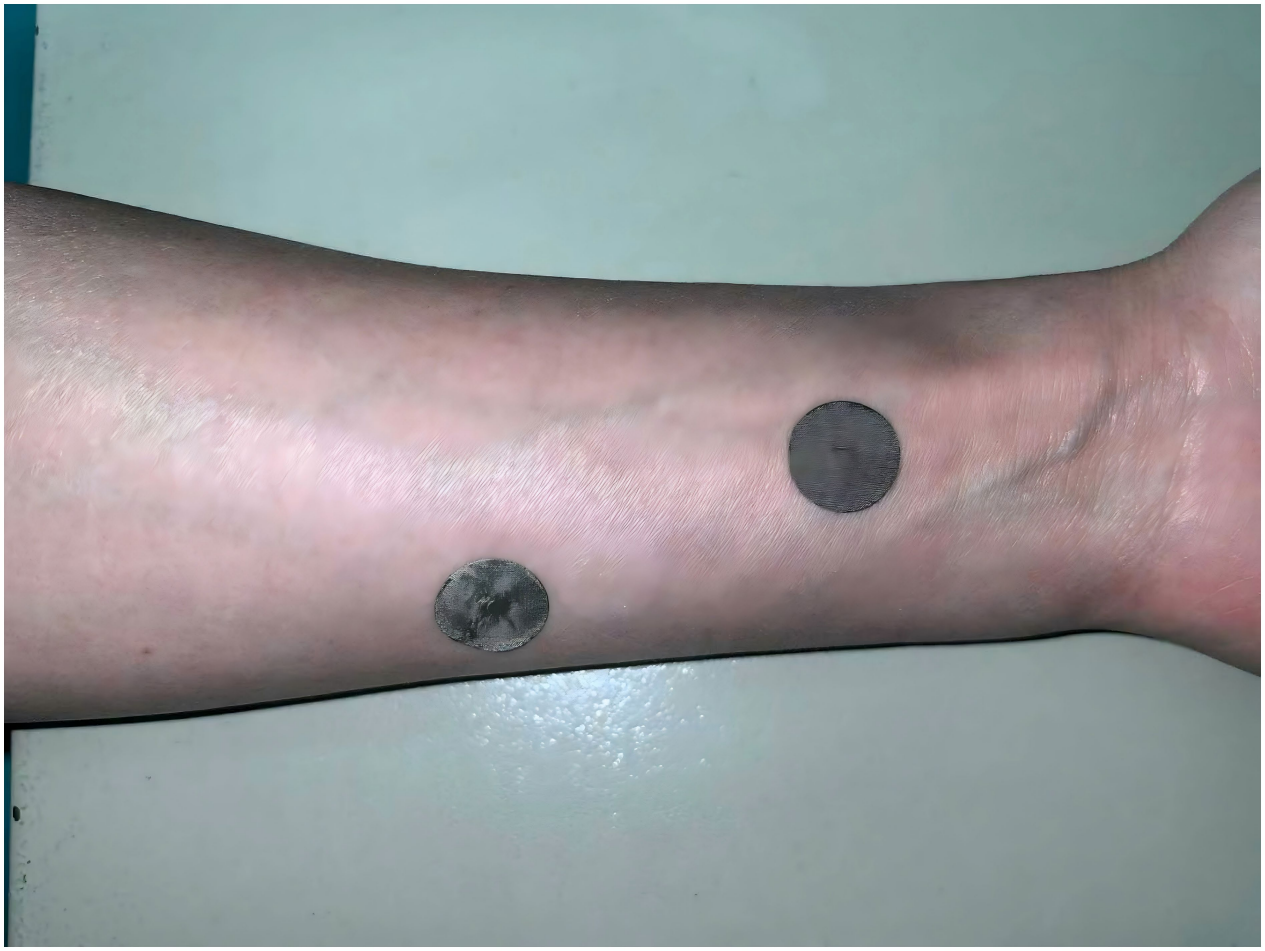
Patients in both groups receive two thumb-tack devices: A real needle (0.25 mm × 2.0 mm) and a sham needle with identical appearance but no needle tip. In Group A, the real device is applied to PC6 point – located three finger breadths proximal to the wrist crease between the palmaris longus and flexor carpi radialis tendons – while the sham device is positioned at a non-acupoint site six finger breadths proximal to the wrist crease and 2 cm ulnarly. Group B receives the inverse configuration, with sham stimulation at PC6 and real stimulation at the non-acupoint site. The sham site maintains a 3 cm radius free of established acupoints to prevent collateral stimulation.

**Figure 4:**
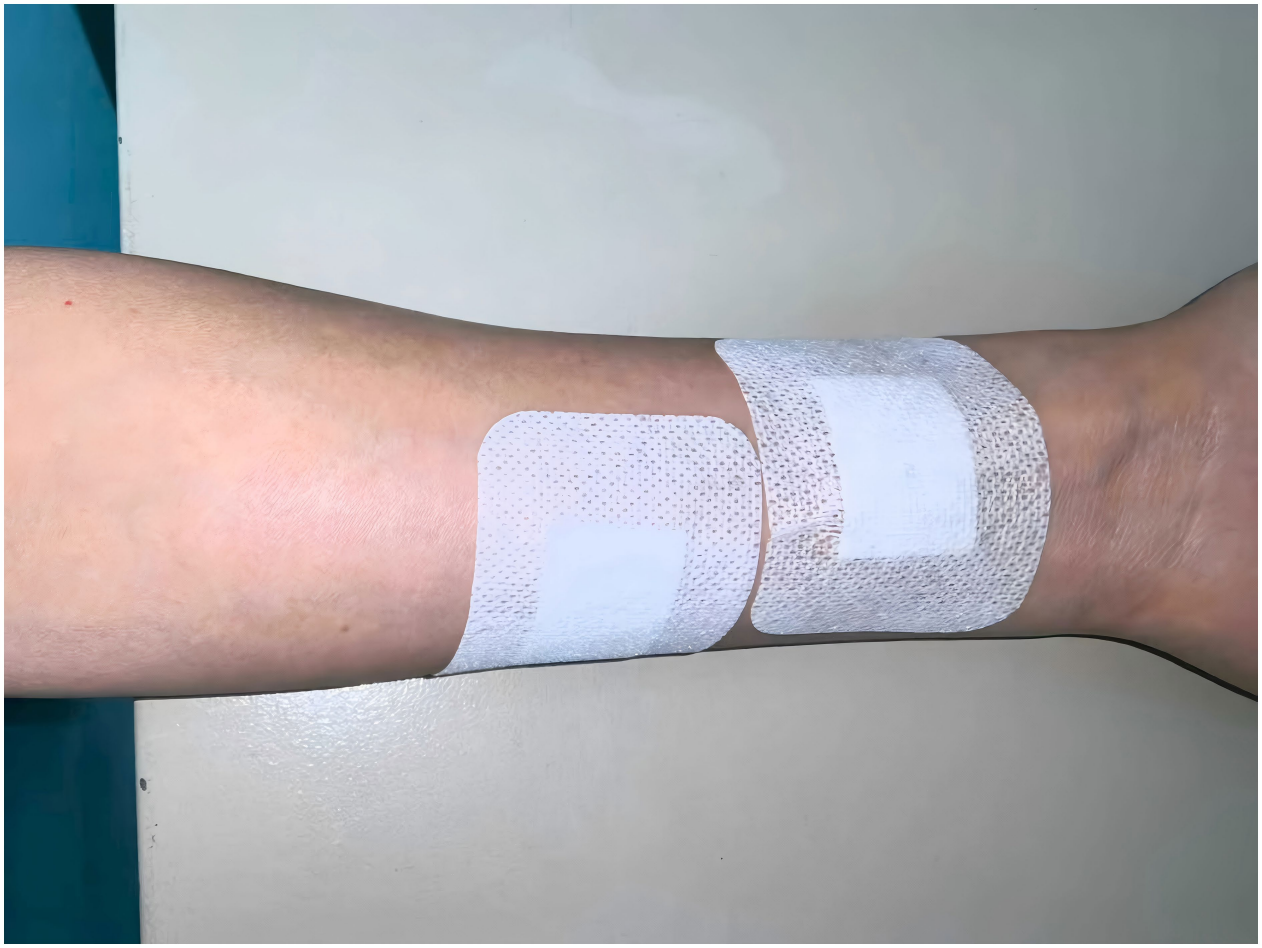
Opaque sterile patches will be applied to both intervention sites to conceal the allocation information.

Upon entering the operating room, the acupuncturist generates a randomization sequence via the Random.org website (https://www.random.org/) to access the corresponding sealed envelope containing group allocation details. Before induction of anesthesia, the acupuncturist applies the assigned thumb-tack devices bilaterally according to the randomized allocation. Both groups receive two thumb-tack devices: a real needle (0.25 mm × 2.0 mm, disposable sterile press needles) and a sham needle with identical appearance but no needle tip. In Group A, the real device is bilaterally applied to the PC6 acupoint – located three finger breadths proximal to the wrist crease between the palmaris longus and flexor carpi radialis tendons – while the sham device is bilaterally positioned at the non-acupoint site six finger breadths proximal to the wrist crease and 2 cm ulnarly. Group B receives the inverse configuration, with sham stimulation bilaterally at PC6 and real stimulation bilaterally at the non-acupoint site. The sham site maintains a 3 cm radius free of established acupoints to prevent collateral stimulation (Figure 3). No deliberate De Qi sensation is elicited during application. Opaque sterile patches will be applied to both intervention sites to conceal the allocation information (Figure 4), with devices remaining *in situ* for 24 postoperative hours. All acupuncture-related procedures are performed in strict accordance with aseptic principles to minimize the risk of infection. Both groups receive standardized PONV prophylaxis per guidelines (18): dexamethasone 5 mg, palonosetron 0.075 mg, propofol target-controlled infusion (TCI) 0.5 μg/mL, and pharyngeal packing.

### Anesthetic management

All patients will undergo standardized preoperative fasting, 8 h for solids, and 3 h for clear fluids. Continuous monitoring will include non-invasive blood pressure, electrocardiogram (ECG), pulse oximetry (SpO_2_), axillary temperature, end-tidal carbon dioxide (EtCO_2_), inspired anesthetic concentrations, and hourly urine output measurement. To enhance external validity and minimize confounding, anesthetic management will be standardized. Anesthesia induction will be achieved with dexamethasone 5 mg IV, propofol TCI 3–4 μg/mL, sufentanil 0.2–0.4 μg/kg, and rocuronium 0.6–0.8 mg/kg. Nasotracheal intubation under direct laryngoscopic guidance initiates mechanical ventilation using a lung-protective strategy: FiO2 40–60%, tidal volume 6 mL/kg predicted body weight, with EtCO_2_ maintained at 35–45 mmHg through respiratory rate adjustment. Maintenance combines propofol TCI 0.5 μg/mL, sevoflurane 1.5–2.5% (minimum alveolar concentration ≥1.0 MAC), remifentanil 0.1–0.2 μg/kg/min, and supplemental rocuronium boluses. Invasive arterial pressure monitoring via radial artery catheterization will be established in the non-dominant upper extremity.

All patients receive intravenous cefuroxime axetil 1.5 g immediately post-induction, with an equivalent prophylactic dose administered 3 h intraoperatively. Axillary temperature maintenance ≥36 °C is ensured using forced-air warming blankets as required. Flurbiprofen 50 mg is administered intravenously 6–7 min prior to surgical skin incision to prevent inflammatory pain. Local infiltration anesthesia with 4% lidocaine containing 1:100,000 epinephrine is applied to both mandibular and maxillary regions. Pharyngeal packing with sterile gauze is implemented to minimize blood ingestion. Controlled hypotension during Le Fort I osteotomy is maintained at a mean arterial pressure (MAP) 50–65 mmHg through combined administration of sevoflurane, metoprolol, and urapidil. Sufentanil 5 μg combining with palonosetron 0.075 mg are administered 30 min prior to surgical closure as preemptive analgesic and antiemetic therapy. Postoperative analgesia was provided via patient-controlled intravenous analgesia (PCIA) using a solution containing sufentanil 100 μg and flurbiprofen 200 mg, diluted with normal saline to a total volume of 100 mL. The pump was programmed with a basal infusion rate of 2 mL/h, a bolus dose of 2 mL, and a lockout interval of 15 min. Neuromuscular blockade reversal with sugammadex is selectively administered based on the patient’s muscle strength recovery. Rescue therapy for refractory PONV consists of intravenous droperidol 1 mg. Supplemental tramadol 100 mg IV is administered if Numerical Rating Scale (NRS; 0 = no pain, 10 = worst pain imaginable) scores exceed 3 points despite three consecutive effective PCIA boluses, with repeat dosing permitted as clinically indicated.

### Data collection and outcome assessment

Following informed consent acquisition, research staff will document baseline parameters including gender, age, height, body weight, body mass index (BMI), history of motion sickness, smoking status, ASA classification, and Apfel risk score (6). Perioperative records will encompass surgical duration, intraoperative fluid balance, controlled hypotension period (defined as MAP <65 mmHg), extubation latency (time from surgery completion to tracheal tube removal), and cumulative dosages of anesthetic agents (propofol, sufentanil, remifentanil).

A designated and trained clinical research specialist will conduct follow-up assessments twice daily (08:00–10:00 and 18:00–20:00) during the initial 24 postoperative hours, evaluating PONV severity, pain intensity, and adverse events. The 12-point PONV assessment scale documents the worst nausea episode per evaluation window, in which 0 = no nausea, 10 = most severe nausea, and 11 = retching/vomiting.

The primary outcome measure of this study is the 24-h incidence of any episode of nausea, vomiting (retching or vomiting) or both (i.e., postoperative nausea and vomiting) postoperatively. PONV is defined as the PONV score ≥1.

Secondary outcomes include: (1) stratified PONV incidence at 0–2 h, 2–6 h, and 6–24 h intervals; (2) peak PONV severity scores during these timeframes; (3) 24-h postoperative QOR-15 score to assess the recovery of patients; (4) the total number of patient-controlled analgesia pump pressed by the patient within 24 h; (5) overall NRS pain score within 24 h; (6) postoperative hospital length of stay; (7) the consumption of tramadol and droperidol.

### Adverse events management

1. Bradycardia: Defined as heart rate <40 bpm or 30% below baseline. Administer intravenous atropine 0.5 mg, repeatable at 5–10 min intervals as clinically indicated.
2. Tachycardia: Defined as heart rate >100 bpm or 30% above baseline. For hypovolemic patients, administer crystalloid solutions; for euvolemic patients, consider esmolol infusion.
3. Hypotension: Systolic blood pressure <80 mmHg or >30% decrease from baseline. Following volume status assessment, implement volume expansion, vasopressor administration, and/or inotropic support.
4. Hypertension: Systolic blood pressure >180 mmHg or >30% increase from baseline. Administer intravenous urapidil.
5. Hypoxia: SpO2 < 90%. Implement supplemental oxygen, deep breathing/coughing, chest physiotherapy, non-invasive ventilation or mechanical ventilation as required.
6. Device-related complications: Needle breakage, intractable pain, local hematoma, infection, or patient intolerance.

All adverse events require detailed documentation and clinical monitoring until resolution.

### Sample size estimation

Based on existing literature (8), PONV incidence in the control group is estimated to be 61% (p_1_ = 0.610). We anticipate that by minimizing risk strategies and acupoint stimulation, we hypothesize a 30% relative reduction in PONV incidence following bilateral orthognathic surgery [p_2_ = p_1_ × (1–0.3) = 0.610 × 0.7 = 0.427]. The 2020 Fourth Consensus Guidelines for PONV established equivalent efficacy between PC6 acupoint stimulation and pharmacological prophylaxis regarding nausea control, vomiting reduction, and rescue antiemetic requirements. Therefore, this trial aims to assess the combinatorial effect of acupuncture, dexamethasone, and palonosetron on PONV incidence reduction in patients undergoing bilateral orthognathic surgery, using a two independent-sample superiority test for sample size calculation. A one-sided superiority test is employed, with a significance level *α* set at 0.05, and Zα being 1.645. The statistical power (confidence level) is 1-*β*, which is 0.8, and Zβ is 0.84. The sample size per group is calculated using the formula:


$$n=\frac{{\left(Z_{\alpha }\sqrt{2\bar{p}(1-\bar{p})}+Z_{\beta }\sqrt{p_{1}(1-p_{1})+p_{2}(1-p_{2})}\right)}^{2}}{{\left(p_{1}-p_{2}\right)}^{2}}$$

The derived minimum sample size per group is 92 (total *N* = 184). Accounting for 10% attrition and implementing stratified randomization with block size 4, the adjusted final enrollment target reaches 104 participants per group (total *N* = 208).

### Statistical analysis

The statistical analysis will be performed with the SPSS 25.0 software. Continuous variables are reported as mean ± standard deviation (SD) or median (interquartile range), while categorical variables are expressed as frequencies or percentages. Parametric data were analyzed using ANOVA or the Kruskal-Wallis H-test, whereas nonparametric comparisons employed chi-square tests (*χ*^2^ tests) or Fisher’s exact probability tests. The primary outcome is the 24-h PONV incidence rate. Superiority testing will assess whether the experimental intervention’s efficacy exceeds that of the control group, derived from literature-derived baseline PONV incidence (control group p₁ = 0.610) and hypothesized 30% relative risk reduction (experimental group p₂ = 0.427). The predefined superiority margin (*δ*) is set as an 18.3% absolute risk reduction (i.e., the incidence in the experimental group must be ≤ the incidence in the control group – δ, where *δ* = 0.183). A single-tailed *Z*-test will be used to compare the incidence difference between the two groups, calculating the risk difference (risk difference, RD) with corresponding 97.5% one-sided confidence interval (equivalent to 95% two-sided CI upper bound). Superiority will be established if the CI upper limit for RD remains below *δ*. Relative risk (RR) with 95% CI will supplement these analyses. Nonparametric alternatives (Fisher’s exact test) will substitute when normal approximation assumptions are violated. Post-hoc exploratory analyses are used to examine heterogeneity in the primary outcome across predefined subgroups, including age <25 years, female sex, history of motion sickness/PONV, smoking status, surgery duration >200 min, and hypotension duration >60 min. Logistic regression is employed to adjust for treatment-covariate interactions of subgroup factors. A *p*-value <0.05 will be considered statistically significant. Except for the superiority test, which employs a one-sided test, all other tests are two-sided.

### Ethics and dissemination plan

The study protocol has undergone tripartite scientific review by the Clinical Research Institute of Peking University Shenzhen Hospital. Four independent clinical experts evaluated the trial’s methodological rigor and operational feasibility. Implementation strictly complies with the Declaration of Helsinki and China’s regulatory requirements for clinical investigations, ensuring ethical integrity and participant safety. Prior to the trial commencement, a comprehensive research plan was meticulously prepared and submitted for rigorous review by the ethics review committee. Following formal approval from the Medical Ethics Committee of Peking University Shenzhen Hospital (approval number: 2025124), the trial achieved prospective registration with the ITMCTR (Trial registration number: ITMCTR2025001713), fulfilling international standards for research transparency.

The participants in this study are patients scheduled for elective surgeries at our institution. On the preoperative day, members of the research team visit each patient at the bedside to provide thorough explanations regarding the objectives and methodology of the study. Prior to obtaining written informed consent, investigators verify participants’ comprehensive understanding of study-related benefits and potential risks. All collected data will use de-identified alphanumeric codes in lieu of personally identifiable information to ensure participant confidentiality during analysis and publication.

Participation in this clinical trial is strictly voluntary. All enrolled subjects retain unconditional rights to withdraw at any stage without compromising their standard perioperative care or incurring therapeutic discrimination. Non-participants and withdrawn cases receive identical evidence-based PONV prophylaxis as per institutional protocols during surgical interventions. The investigative team assumes full financial responsibility for managing study-related adverse events, including emergency medical expenses and extended hospitalization costs attributable to trial participation.

Following trial completion, de-identified research outcomes will be disseminated through international peer-reviewed journals to advance evidence-based management of post-orthognathic PONV. All confidentiality-protected participant data (including demographic records and medical histories) will be permanently excluded from dissemination materials in compliance with HIPAA-compliant standards. This protocol strictly adheres to ethical knowledge generation principles, maintaining equilibrium between scientific transparency and individual privacy preservation throughout the knowledge translation process.

## Discussion

In recent years, due to the rising patients’ expectations for improved quality of life and the advancement of Virtual Surgical Planning techniques, which can precisely simulate aesthetic outcomes, an increasing number of patients have actively requested to undergo orthognathic surgery (10). Notwithstanding inspirational advances in the field, PONV persists as a critical determinant of postoperative recovery trajectories and prolonged hospitalization in orthognathic patients. Severe vomiting may threaten the life safety of patients. Notably, patients demonstrate greater tolerance thresholds for postoperative pain compared to PONV-related distress (21, 22). Compared with pain, PONV prophylaxis remains constrained by limited pharmacological options.

Postoperative pain can be effectively relieved by drugs and nerve blocks, but once PONV occurs, the intraoperative prophylactic agents are ineffective as remedial treatment. Notably, dexamethasone’s antiemetic efficacy necessitates early intraoperative administration to exert an anti-inflammatory effect (23), whereas propofol-based total intravenous anesthesia (TIVA) is restricted to the intraoperative phase. The treatment options for PONV are very limited, so prevention is the focus. Effective prevention can significantly improve patients’ perioperative experience.

The precise pathogenesis of PONV remains incompletely elucidated, with current evidence suggesting multifactorial etiology involving distinct neurobiological pathways (24). Monotherapy targeting single neurotransmitter systems demonstrates limited clinical efficacy due to partial pathway blockade. Current consensus guidelines advocate multimodal prophylaxis combining two or more mechanistically distinct antiemetics for high-risk populations, with dexamethasone plus 5-HT3 receptor antagonists constituting the evidence-based cornerstone regimen (3). It is well known that the type of surgery is a crucial risk factor for PONV. Procedures involving laparoscopic surgery, weight loss surgery, gynecological surgery, and cholecystectomy are well-established risk amplifiers. Currently, most clinical studies on PONV revolve around these types of surgeries (25–30). Orthognathic surgery’s classification remains contentious: the 2007 PONV Management Consensus Guidelines designated it high-risk (31), whereas subsequent 2014/2020 updates rescinded this designation (3, 32). However, even with a combined treatment regimen, the incidence of postoperative nausea was as high as 50% in patients with orthognathic surgery, and even higher in patients with bimaxillary surgery (9). Therefore, in patients undergoing bimaxillary surgery, it may be beneficial to add a third adjunctive preventive measure, namely preoperative intradermal acupuncture, to the baseline dual antiemetic regimen of dexamethasone plus palonosetron (8). Acupuncture, transcutaneous electrical nerve stimulation (TENS), or capsaicin stimulation of PC6/ST36 have been clearly demonstrated to prevent PONV, as non-inferior to conventional antiemetics of metoclopramide, cyproheptadine, promethazine, ondansetron, and dexamethasone (33–35). Practical constraints limit intraoperative acupuncture protocols – most studies employ transient perioperative stimulation without sustained postoperative retention. Once patients leave the operating room, all stimuli are withdrawn. However, postoperative facial swelling and swallowing blood in orthognathic surgery patients remain significant risk factors for PONV. This temporal discontinuity raises the clinical question of whether intraoperative-only acupoint stimulation still effectively prevents PONV requires further clinical research to confirm.

The intradermal needle, a thumb-tack shaped medical device, is subcutaneously implanted to deliver sustained acupoint stimulation through localized mechanical activation. This modality provides continuous regulation of meridian function and qi-blood balance through controlled dermal neuromodulation (36, 37). Clinical evidence supports extended indwelling periods (up to 72 h) with minimal discomfort and unrestricted patient mobility, contributing to its widespread adoption in contemporary practice (38–40). The superficial anatomical placement of PC6 allows precise needle insertion using microfilament devices, effectively avoiding trauma to critical neurovascular structures including the median nerve and radial artery in the wrist region.

To optimize blinding integrity, a forearm region devoid of established acupoints within a 3 cm radius is selected as the sham stimulation site. Participants in the sham group receive superficial dermal stimulation mimicking needle insertion trauma using identical sterile devices. Opaque surgical dressings are symmetrically applied to all intervention sites to preserve allocation concealment. This protocol ensures triple blinding: neither participants, anesthesiologists, nor outcome assessors can visually or tactilely discern active versus sham needle placement.

## Limitations

Maintaining double-blinding integrity presents the primary methodological challenge in acupuncture trials. Standard acupuncture protocols typically recommend manual compression of intradermal needles for 1–2 min every 3–4 h during indwelling periods to optimize mechanical transduction of acupoint stimulation and augment therapeutic efficacy through sustained neuromodulatory response (41).

To preserve blinding integrity, our protocol intentionally avoids eliciting De Qi sensation during needle insertion and discourages postoperative manual acupoint stimulation. While this methodology safeguards allocation concealment, it may attenuate the therapeutic efficacy of intradermal needles in preventing PONV. Furthermore, although sham needle placement at non-acupoint sites maintains anatomical distance (>3 cm) from established acupoints, the inherent mechanical nociception from cutaneous penetration introduces confounding somatosensory input. This unintended physiological stimulus could theoretically modulate emetic pathways in the control group participants, potentially inflating Type II statistical errors through PONV incidence rate convergence between study arms.
